# Comprehensive molecular characterization of human adipocytes reveals a transient brown phenotype

**DOI:** 10.1186/s12967-015-0480-6

**Published:** 2015-04-30

**Authors:** Andrea Guennoun, Melissa Kazantzis, Remy Thomas, Martin Wabitsch, Daniel Tews, Konduru Seetharama Sastry, Mouaadh Abdelkarim, Vladimir Zilberfarb, Arthur Donny Strosberg, Lotfi Chouchane

**Affiliations:** Laboratory of Genetic Medicine & Immunology, Weill Cornell Medical College in Qatar, P.O. Box 24144, Doha, Qatar; Center for Diabetes and Metabolic Diseases, The Scripps Research Institute, Florida, USA; Department of Paediatrics and Adolescent Medicine, Division of Pediatric Endocrinology and Diabetology, Ulm, Germany; Department of Physiology, King Saud University, Riyadh, Saudi Arabia; Institut Cochin INSERM U1016, Université Paris 7‐Denis‐Diderot, Paris, France; Department of Infectology, The Scripps Research Institute‐Florida, Jupiter, FL USA

**Keywords:** Obesity, SGBS cells, Human adipocytes, Non-shivering thermogenesis, BAT, WAT, Beige adipocytes

## Abstract

**Background:**

Functional brown adipose tissue (BAT), involved in energy expenditure, has recently been detected in substantial amounts in adults. Formerly overlooked BAT has now become an attractive anti-obesity target.

**Methods and results:**

Molecular characterization of human brown and white adipocytes, using a myriad of techniques including high-throughput RNA sequencing and functional assays, showed that PAZ6 and SW872 cells exhibit classical molecular and phenotypic markers of brown and white adipocytes, respectively. However, the pre-adipocyte cell line SGBS presents a versatile phenotype. A transit expression of classical brown markers such as UCP1 and PPARγ peaked and declined at day 28 post-differentiation initiation. Conversely, white adipocyte markers, including Tcf21, showed reciprocal behavior. Interestingly, leptin levels peaked at day 28 whereas the highest adiponectin mRNA levels were detected at day 14 of differentiation. Phenotypic analysis of the abundance and shape of lipid droplets were consistent with the molecular patterns. Accordingly, the oxidative capacity of SGBS adipocytes peaked on differentiation day 14 and declined progressively towards differentiation day 28.

**Conclusions:**

Our studies have unveiled a new phenotype of human adipocytes, providing a tool to identify molecular gene expression patterns and pathways involved in the conversion between white and brown adipocytes.

**Electronic supplementary material:**

The online version of this article (doi:10.1186/s12967-015-0480-6) contains supplementary material, which is available to authorized users.

## Background

Over the last two decades the prevalence of obesity has reached epidemic levels worldwide. In 2008, studies estimated that around 1.5 billion adults had a BMI of 25 kg/m2 or greater, and out of these 205 million men and 297 million women had a BMI of 30 kg/m2 and were classified as obese [1]. Secondary major health risks, such as but not limited to hypertension, insulin resistance, type 2 diabetes (T2D) and cardiovascular diseases are highly associated with obesity and are seen as well in increasing numbers of adults worldwide [2,3].

Obesity most commonly results if the energy intake (e.g. through energy-dense food) exceeds energy expenditure, and as a direct consequence the overall energy homeostasis is imbalanced. This excess energy is stored in the body in form of white fat, [4] which also acts as an endocrine organ by releasing adipokines and cytokines into the bloodstream [5]. The adipocyte-derived hormone leptin e.g. which regulates energy intake and appetite was reportedly found at elevated levels in the bloodstream of obese subjects and is positively correlated with BMI [6].

While some of the functions and implications of white adipose tissue (WAT) have been discovered and are investigated, understanding of the underlying biology is far from comprehensive or complete. In addition to WAT, functional BAT in adults has been discovered through radiological detection by several research groups [7-9] and has recently become a promising therapeutic target due to its ability to dissipate energy in form of heat, through a process called non-shivering thermogenesis (or uncoupled respiration). Surprisingly, only 50 g of BAT are believed to be sufficient to burn 20% of the overall average energy intake of a healthy adult [10]. Moreover, some studies propose that the amount of BAT in humans is inversely associated with BMI, i.e. healthy, normal-weight subjects bear a greater amount than obese or overweight individuals [11-13].

Therefore, the idea of enriching BAT or converting WAT into functional BAT has become attractive and many efforts have been started to investigate the possibilities further. Promoting BAT-like features in white adipocytes, known as “browning”, leads to the remodeling of WAT as energy storage into an energy dissipation site. Experimental protocols have been pursued that interfere either with the PPARγ pathway by SirT1-dependent deacetylation [14], or in the p38 mitogen-activated protein kinase and extracellular signal-related kinase signaling pathways by a hormone called irisin [15]. It has been suggested that this “transdifferentiation” could be an inherent property of white adipocytes and may not be due to a distinct cell type with this predisposition [16]. However, the identification of brown-in-white/brite/beige adipocytes has led to the assumption that a small proportion of inducible adipocytes is naturally present in some classical WAT depots [17]. These beige cells can be induced by cold or β-adrenergic stimulation and can be observed as UCP1-positive islets [18].

SGBS cells [19] are widely used as a model for white adipocytes. However, our data suggest that the cell line harbors an inducible phenotype that changes throughout the differentiation stage. Day 14 displays multiple characteristics of brown fat cells whereas day 28 represents a rather white phenotype. Many reports have recently suggested that the traditional classification of adipocytes is not exclusive and the existence of beige/brite adipocytes has been shown.

Studies aimed at pinpointing the mechanisms involved in the conversion between white and brown/beige/brite human adipocytes and metabolic characterizations are largely dependent on the use of *in-vitr*o cell systems given the limited access to human subjects and range of human procedures that can be performed for obvious ethical reasons.

Thus, we were aiming to elucidate the unique nature of SGBS cells characterized by displaying transient features of brown adipocytes and eventually progressing to a white phenotype. In depth functional and phenotypic analysis of differentiated SGBS cells at both distinct stages and comparison with brown PAZ6 and white SW872 adipocytes allowed the study of molecular gene expression patterns and pathways involved in the conversion between white and brown adipocytes. This knowledge will be of importance for studies aiming to increase brown fat depots in order to increase energy expenditure in obese subjects with the ultimate goal of weight reduction.

## Methods

### Cell lines/cell culturing

The brown pre-adipocyte cell line PAZ6 was kindly gifted by Dr. D. Strosberg [20] and SGBS cells were kindly provided by Dr. M. Wabitsch [19]. SGBS pre-adipocytes were grown in DMEM/F12 medium supplemented with 10% fetal calf serum (FCS), 1.7 mM panthotenat, 3.3 mM biotin and 1% penicillin/streptomycin (pen-strep). Differentiation was performed at full confluency. Differentiation medium consisted of DMEM/F12 supplemented with transferrin (0.01 mg/ml), insulin (20nM), cortisol (100nM), and triiodothyronine (T3, 0.2 nM). For the initial four days, the differentiation medium was additionally supplemented with dexamethasone (25nM), IBMX (250 μM) and rosiglitazone (2 μM).

PAZ6 pre-adipocytes were cultured in DMEM/F12 medium enriched with 8% FCS, 15 mM Hepes and 1% pen-strep. At full confluency, differentiation was propagated by addition of 5% FCS, 15 mM Hepes, 33 μM biotin, 17 μM panthotenat, 1nM T3, 100nM dexamethasone, 1 μM rosiglitazone and 1% pen-strep to DMEM/F12 medium. Additionally, for the initial four days 0.25 mM IBMX was added to the differentiation medium.

Human SW872 pre-adipocytes were purchased from ATCC and cultured in DMEM/F12 medium enriched with 8% FCS, 15 mM Hepes and 1% pen-strep. At 80 – 100% confluency, oleic acid was added at a final concentration of 100 μM to initiate differentiation.

All culture and differentiation media were changed every two days and cell cultures were carried out at 37°C under 5% CO_2_.

For cold exposure of SGBS cells, we transferred fully differentiated cells at D14 to an incubator set to 30°C and 5% CO_2_ for 4-6 h prior to subsequent RNA isolation. For staining purposes, we kept the culture plates for additional 10 days until day 14 at 30°C and 5% CO_2_ after the first four days in 37°C.

### RNA isolation and cDNA synthesis

RNA was isolated using Qiagen RNeasy lipid tissue mini kit and manufacturer’s instructions were followed. The concentration of obtained total RNA was measured using Nanodrop reading. 500 ng of RNA was reversely transcribed in cDNA using GoScript reverse transcriptase (Promega).

### Quantitative RT-PCR

qPCR reactions were carried out in triplicates on an ABI 7500 cycler using GoTaq qPCR mastermix (Promega). Expression values were calculated as 2^-ΔCT^ using HPRT as reference. Primer sequences are listed in the supplement (Additional file 1: Table S1).

#### Western blotting

Cells were lyzed in RIPA buffer and separated by SDS-PAGE. Immunoblotting was carried out with anti-UCP1 antibodies (Abcam, dilution 1:500) and as loading control β-actin protein levels were assessed (Abcam, dilution 1:2500). Secondary horseradish-peroxidase-conjugated anti-mouse antibodies were purchased from Sigma and used at a dilution of 1:5000.

### RNAsequencing

Total RNA was isolated with the RNeasy lipid tissue mini kit (Qiagen) and the manufacturer’s instructions were followed. Purified total RNA was subjected to deep sequencing analysis. Initially, isolated RNA was quantified using Agilent Bioanalyzer 2100 with the RNA integrity number greater than 8.0 before Illumina Genome Analyzer (GA) sequencing. RNA sequencing (RNAseq) was performed using the Illumina Genome Analyzer to measure mRNA expression levels from seven human cell line samples. Typically, 2–4 ug total RNA were used in library construction. Total RNA was reverse transcribed to double-stranded cDNA, digested with NlaIII and ligated to an Illumina specific adapter containing a recognition site of MmeI. Following MmeI digestion, a second Illumina adapter, containing a 2-bp degenerate 3’ overhang was ligated. The obtained sequences were aligned onto human RefSeq database (ftp://ftp.ncbi.nih.gov/refseq) using SOAP software [21]. Only uniquely mapped sequences to RefSeq genes were kept for subsequent analysis. For each mRNA sample, we generated on average a total 26 M raw reads. On average, 84% of the reads matched known genes, and ~16,000 genes had expression level data. Raw data are publicly available through the NCBI GEO database [GSE63190].

### Gene expression analysis

Gene expression values were summed from normalized RNAseq reads. Hierarchical clustering of gene expression was based on Speerman rank correlation coefficient between gene expression values of each of the three cell lines using complete linkage for agglomeration. As an approach to identify differentially expressed genes (DEGs), we applied the classical single *t*-test.

### Gene ontology analysis

Gene ontology (GO) enrichment analysis provides all GO terms that are significantly enriched in a list of DEGs by aligning GO terms to the database (http://www.geneontology.org/) and KEGG pathways (http://www.genome.jp/kegg/). Lists of differentially expressed genes were clustered and calculate by Cluster and Java Treeview software. Transcripts, which had missing values in at least one experiment, were removed from the analysis. Pathway and gene interaction analysis was performed with Ingenuity Pathway analysis software.

### Oil Red staining

Adherent pre-adipocytes and differentiated adipocytes were fixed with 4% paraformaldehyde for 30 min at room temperature (RT). After one washing step with PBS, cell layers were washed with 60% isopropanol once. Oil Red solution was added for 5 min at RT and rinsed off with diH_2_0 until clear. Stained adipocytes were assessed under a light microscope and images were analyzed using Adobe Photoshop software.

### Immunofluorescence microscopy

For UCP1 staining, adherent pre-adipocytes and differentiated adipocytes were cultured in glass bottom slides and fixed with 4% paraformaldehyde for 30 min at RT. Next, cells were blocked with 2% BSA for one hour in order to prevent unspecific binding and incubated overnight with anti-UCP1 antibody (Abcam, diluted 1:250) at 4°C. Secondary antibody incubation was carried out in the dark at RT for one hour with anti-mouse Alexa 647 (Life technologies, diluted 1:500) and in the presence of Mitotracker (Life technologies, 10nM final concentration).

For lipidtox stainings of the lipid droplets, cells were fixed with 4% paraformaldehyde for 30 min at RT. After one washing step with PBS, cells were co-stained with Lipidtox red (Invitrogen, diluted 1:200) and Mitotracker green (Life technologies, 10nM final concentration) for 30 min in the dark at 37°C.

Nuclei were stained using BlueNuc stain (Life technologies). Cell monolayers were examined using a digital EVOS f1 microscope equipped with an AMG camera. Digital images were processed with Photoshop CS6 (Adobe Systems, CA, USA).

### Expression of mitochondrial respiratory complexes I, II, III, IV and V

The mitochondrial respiratory complexes analysis was performed using the Human Oxidative Phosphorylation (OXPHOS) Magnetic Bead Panel (Cat. # H0XPSMAG-16K, EMD Millipore, Massachusetts, USA). The immunoassay was carried out in duplicates according to the manufacturer instructions, using antibody-coated fluorescent beads. Antigen bound beads were quantified in the Luminex 200 instrument. Complexes I, II, III, IV and V fluorescent units (FU) were normalized to the nucleus-encoded mitochondrial protein nicotinamide nucleotide transhydrogenase (NNT).

### Quantification of metabolic rates and glycolysis in PAZ6, SGBS and SW872 cells using the Sea horse XF flux analyzer

Oxygen consumption rate (OCR) and extracellular acidification rate (ECAR) from PAZ6 and SGBS adipocytes were measured using the SeaHorse flux analyzer XF96 (Seahorse Biosciences, North Billerica, MA). The PAZ6 or SGBS pre-adipocytes were plated on SeaHorse cell culture plates at 5x10^4^ cells/well to achieve confluence and differentiated for 14 or 28 days. Prior to the assay, the growing media was replaced by unbuffered DMEM containing 2.5 mM glucose and 5 mM pyruvate (Gibco #12800-017, pH = 7.4 at 37 C). Fatty acids (100 μM) were added in some cases, where indicated.

Basal, uncoupled and maximal respiration rates were calculated upon subtraction of the non-mitochondrial oxygen consumption obtained at the end of each assay by the addition of rotenone (4 μM) and myxothiazol (1 μM). ECAR, indicative of glycolytic capacity was also obtained.

#### Statistical analysis

The data presented in the line and column charts are the mean ± SEM from multiple determinations. Data analysis was carried out in Prism 6 (Graph Pad). Statistical significance was determined by t-tests for comparisons between pre-adipocytes versus mature adipocytes and 2-way ANOVA followed by Tukey multiple comparisons test for all other comparisons including 3 groups or more.

## Results

### Comparative analysis of three human adipocyte cell lines

#### The unique human brown adipocyte cell line PAZ6 accumulates multiple small lipid droplets upon differentiation and expresses peri-nuclear and cytoplasmatic UCP1 at high levels

**Figure 1:**
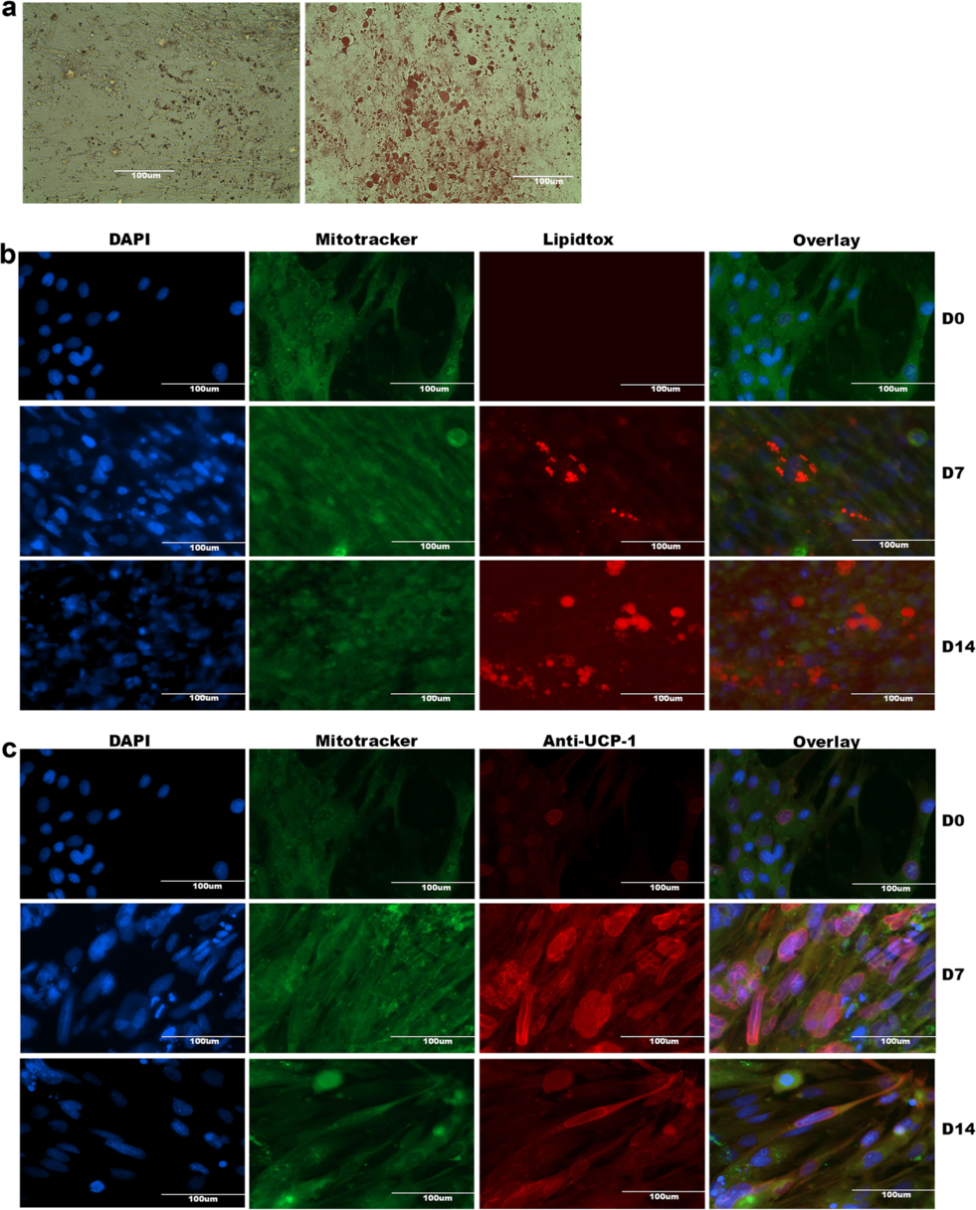
Human brown PAZ6 adipocytes accumulate multiple small lipid droplets upon differentiation and express peri-nuclear and cytoplasmatic UCP1 at high levels. PAZ6 cells were differentiated for 14 days and cell monolayers were stained and examined by microscopy. **(a)** Oil Red staining was carried out as described above and the presence of stained lipid droplets at D7 (left) and D14 (right) was assessed at a magnification of 20×. (**b** and **c**) Premature and differentiated PAZ6 cells were co-stained with mitotracker (green) to test the abundance of mitochondria, lipidtox (red, in **b**) for neutral lipid droplets or anti-UCP1 antibodies (red, in **c**) and DAPI (blue) to visualize nuclei. Single-channel images were overlayed and processed by Photoshop software. All scale bars are reported.

We first characterized both undifferentiated and differentiated human PAZ6 adipocytes and we conducted Oil Red and fluorescence staining experiments of fixed cells, grown in monolayers. Images of PAZ6 were taken on Day 0 (D0), D7 and D14. Initial lipid droplet formation was visible seven days after differentiation initiation and the presence of neutral lipids was confirmed by Oil Red (Figure 1a) and Lipidtox (Figure 1b) staining. Notably, as expected, multiple small lipid droplets consistent with their brown adipocyte phenotype were observed in mature PAZ6 cells. In addition, staining with anti-UCP1 antibody revealed increasing expression of UCP1 protein during the maturation process of PAZ6 cells until D14 (Figure 1c). Co-staining with Mitotracker dye revealed abundance of mitochondria along with the increased expression of UCP-1 in differentiated PAZ6 adipocytes as compared to PAZ6 pre-adipocytes (Figure 1b and c). This confirmed co-localization of UCP1 and mitochondria.

**Figure 2:**
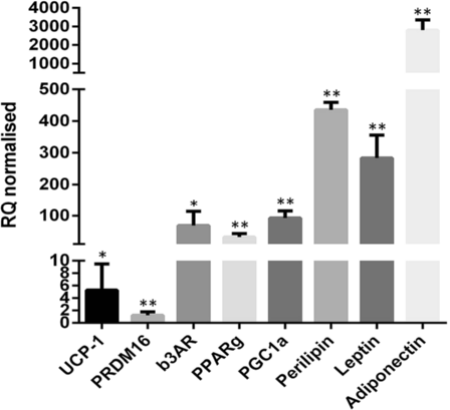
Differentiated PAZ6 cells overexpress adipokines and brown adipocyte markers. PAZ6 cells were cultured in monolayers and differentiated for 14 days. RNA was isolated using the Qiagen lipid tissue mini kit and cDNA synthesis was carried out. Quantitative real-time PCR was performed by SYBR green detection method and values are reported as RQ normalized referring to the relative quantification compared to HPRT expression and normalized for D0 baseline expression levels for each gene. All experiments were done in triplicates and data are derived from at least three independent experiments. (* p <0.01, ** p < 0.001).

Next, we assessed the molecular expression levels of adipocyte markers in pre-mature and differentiated human PAZ6 cells by quantitative real-time RT-PCR. As expected, known brown adipocyte markers such as PGC1α, PRDM16, PPARγ and beta3-adrenergic receptor (b3AR) were found to be up-regulated at D14 after initiating the differentiation process (Figure 2). Importantly, up-regulation of the BAT-defining marker UCP1 was confirmed and consistent with immunofluorescent detection as shown in Figure 1c. In addition, common adipocyte markers such as leptin, adiponectin and perilipin were highly up-regulated in mature PAZ6 cells and underlined the formation and presence of neutral lipid droplets.

#### Differentiated SW872 adipocytes depict a high abundance of lipid droplets but no UCP1 expression

**Figure 3:**
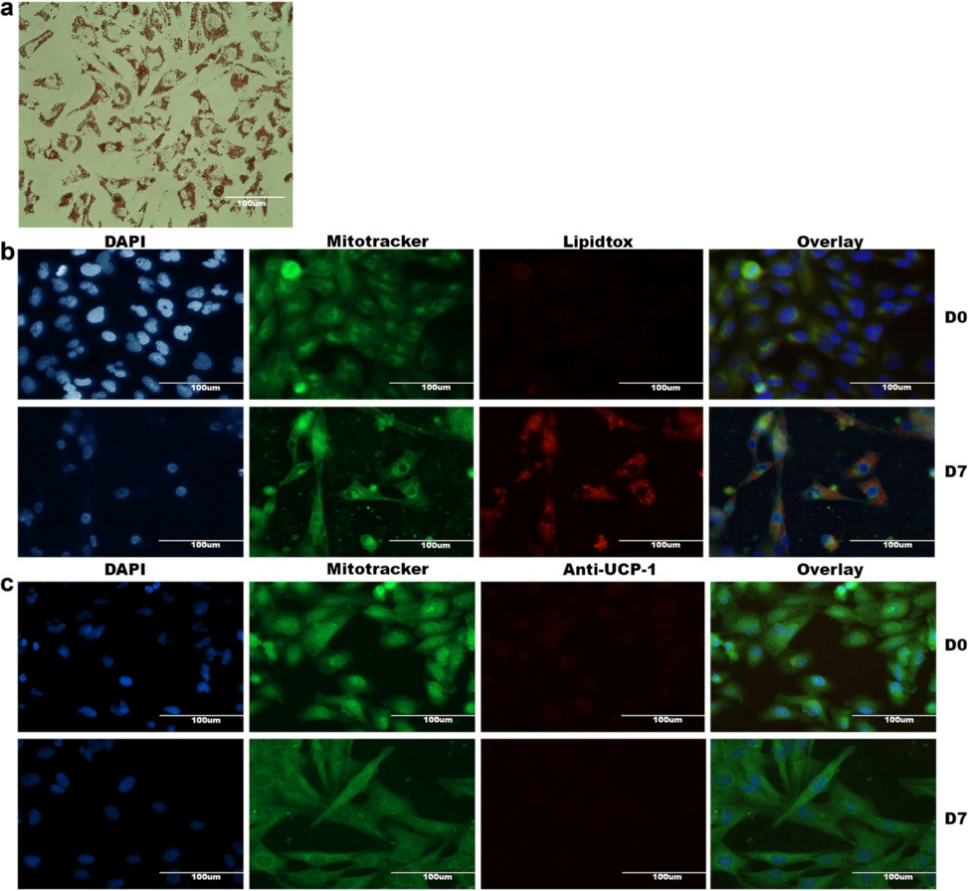
Differentiated SW872 adipocytes depict a high abundance of lipid droplets but no UCP1 expression. **(a)** Oil Red staining was carried out as described above and the presence of stained lipid droplets at D7 was assessed at a magnification of 20×. (**b** and **c**) Premature and differentiated SW872 cells were co-stained with mitotracker (green) to test the abundance of mitochondria, lipidtox (red, in **b**) for neutral lipid droplets or anti-UCP1 antibodies (red, in **c**) and DAPI (blue) to visualize nuclei. Single-channel images were overlayed and processed by Photoshop software. All scale bars are reported.

We then assessed the differentiation potential of SW872 adipocytes by observing the formation of lipid droplets. Interestingly, as opposed to PAZ6 and SGBS cells, 100% of SW872 were differentiated after 7 days of culture and the phenotype did not differ from the one observed at D14. We therefore considered D7 as the final stage of differentiation in SW872 cells and performed subsequent experiments at D7. We confirmed full differentiation by Oil Red (Figure 3a) and fluorescence staining with Lipidtox (Figure 3b). In addition, as shown in Figure 3c, we performed staining with anti-UCP1 antibodies. We did not, however, detect expression of UCP1 in fully differentiated SW872 cells at D7 and no remarkable increase in the abundance of mitochondria from D0 to D7 was observed, as reflected by mitotracker staining (Figure 3b and c).

#### Human SGBS adipocytes display features of white and brown adipocytes, respectively in a time-dependent manner

**Figure 4:**
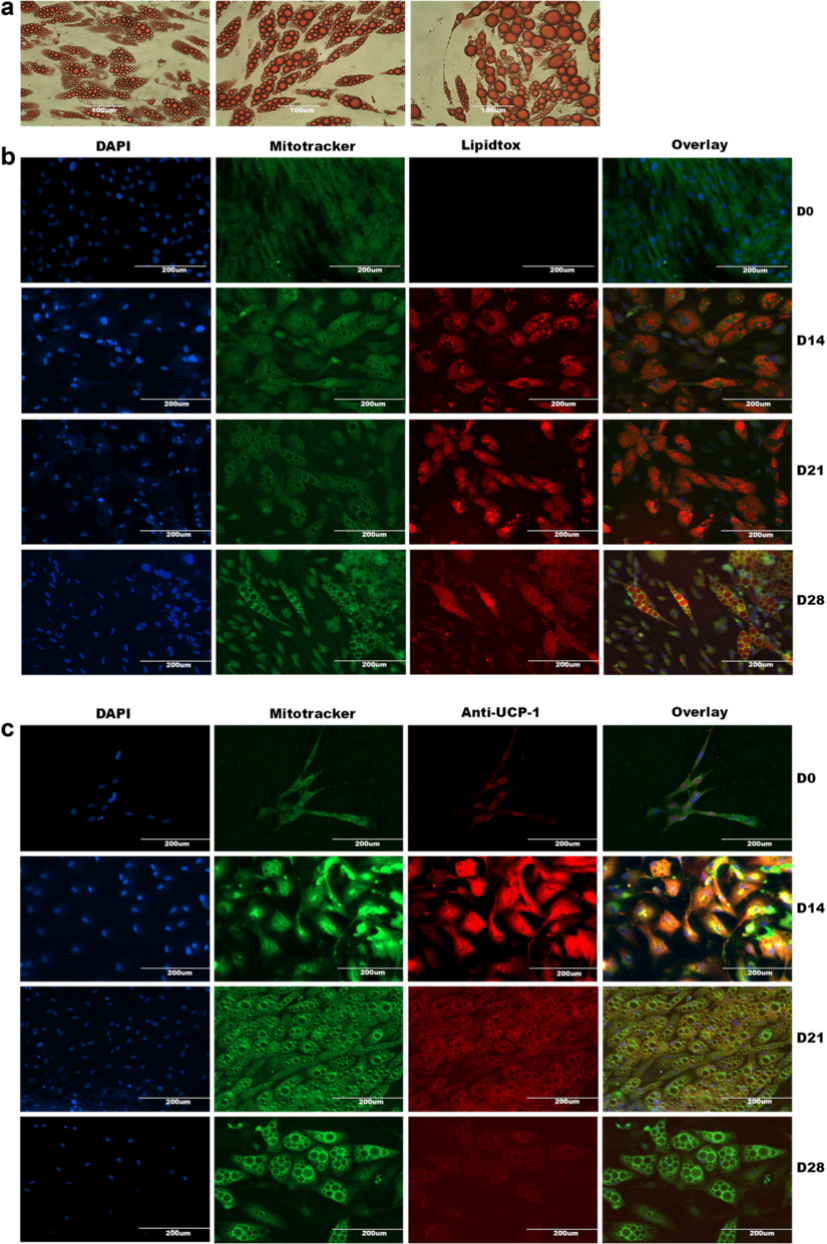
Human SGBS adipocytes display features of white and brown adipocytes respectively. (**a**) Oil Red staining was carried out as described above and the presence of stained lipid droplets at D14, D21 and D28 was assessed at a magnification of 20×. (**b** and **c**) Premature and differentiated SGBS cells were co-stained with mitotracker (green) to test the abundance of mitochondria, lipidtox (red, in **b**) for neutral lipid droplets or anti-UCP1 antibodies (red, in **c**) and DAPI (blue) to visualize nuclei. Single-channel images were overlayed and processed by Photoshop software. All scale bars are reported.

Lastly, we cultured and differentiated SGBS adipocytes up to D14 and observed the formation, abundance and size of lipid droplets. We noted a rather brownish phenotype of mature SGBS cells as characterized by multiple small lipid droplets and decided to keep the culture for an additional two weeks up to D28. Interestingly, and to our surprise, the size and number of lipid droplets changed over the course of four weeks as shown in Figure 4a and b. D14 as initially mentioned was represented by multiple small lipid droplets which increased remarkably in size, decreased proportionally in number up to D28 and exemplified a mature white adipocyte phenotype.

### Further elucidation of the versatile, inducible phenotype of human SGBS cells

We confirmed the findings derived from Oil Red staining in SGBS cells by lipidtox fluorescent imaging and detected remarkable changes in lipid droplet size and number as shown before (Figure 4b). We then tested the expression levels of UCP1 protein in order to comprehend whether D14 truly represented a rather brown and D28 a white adipocyte phenotype. Immunofluorescent images revealed a peak in UCP1 expression at D14 and a decline up to D28 (Figure 4c). Notable changes in the abundance of mitochondria could not be seen and expression levels of mitochondrial protein stained by mitotracker were constant (Figure 4b and c).

#### Molecular analysis of adipokines expression in undifferentiated and mature SGBS cells confirms phenotypic findings

**Figure 5:**
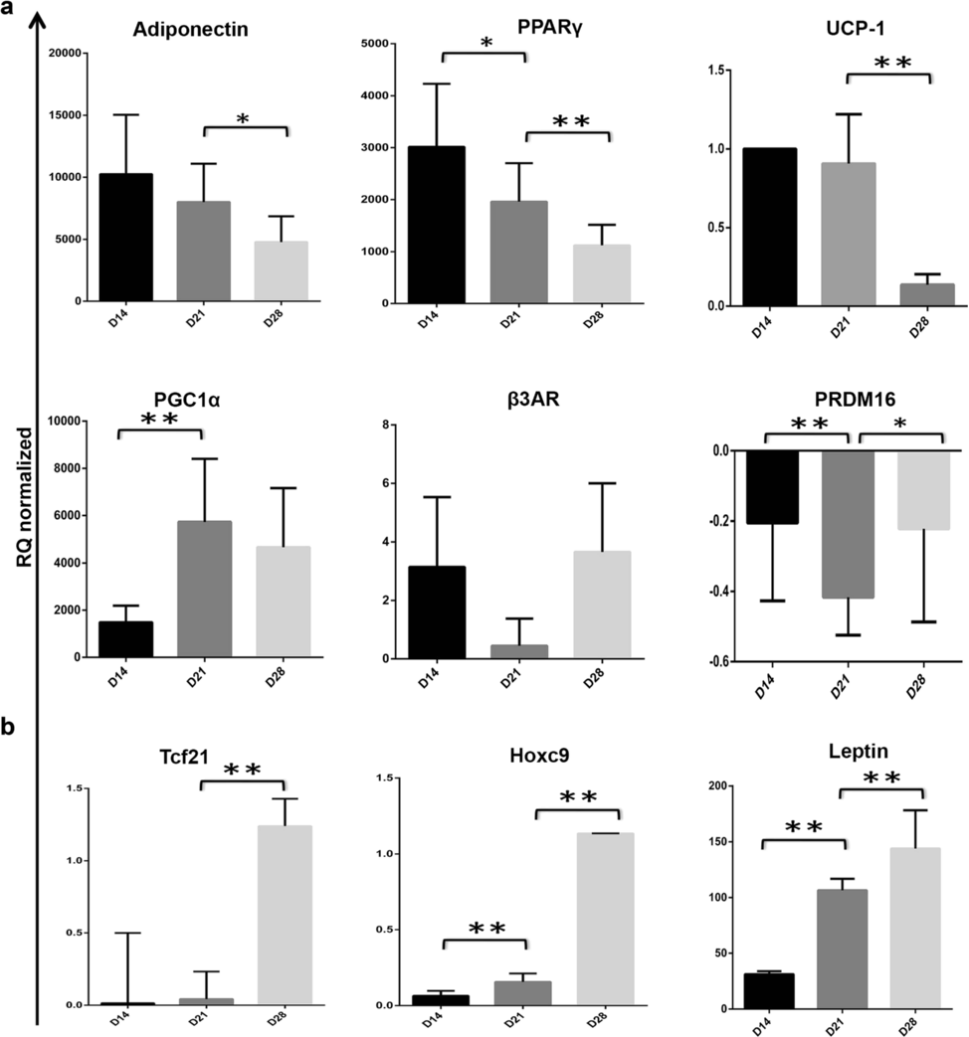
Molecular analysis of adipokine expression in undifferentiated and mature SGBS cells confirms phenotypic findings. SGBS cells were cultured in monolayers and upon reaching confluency, cells were differentiated for 28 days. RNA was isolated using the Qiagen lipid tissue mini kit and cDNA synthesis was carried out. Quantitative real-time PCR was performed by SYBR green detection method and values are reported as RQ normalized referring to the relative quantification compared to HPRT expression and normalized for D0 baseline expression levels for each gene. All experiments were done in triplicates and data are derived from at least three independent experiments. **(a)** Gene expression of markers of brown adipocytes and general fat cell markers were assessed. **(b)** In order to elucidate the versatile phenotype of SGBS cells, expression levels of white adipocyte markers were tested. All experiments were done in triplicates and data are derived from at least three independent experiments. (* p <0.01, ** p < 0.001).

In order to get deeper insight into the molecular expression levels of key molecules we performed quantitative RT-PCR. The expression levels of brown adipocyte markers, such as UCP1, PGC1α and PPARγ decreased on D28 after peaking at D14 (PPARγ or UCP1) or D21 (PGC1α). PRDM16 and b3AR expression levels were similar in their expression pattern with a low level at D21 and comparable expression levels at D14 and D28 (Figure 5). Interestingly, key markers of white adipocyte markers such as Hoxc9, Tcf21 or the pan-adipocyte marker leptin displayed increasing expression level on the course of 4 weeks and peaked at D28 (Figure 5). Lastly, a highly significant down-regulation of adiponectin from D14 to D28 was observed (Figure 5).

#### Direct comparison of UCP1 protein levels of PAZ6 and SGBS adipocytes confirms the transient BAT-like phenotype of SGBS cells

**Figure 6:**
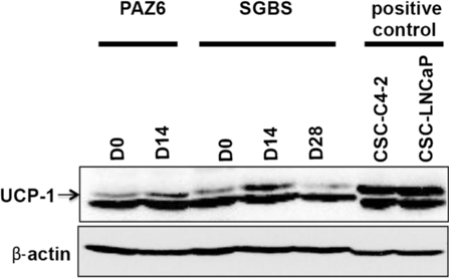
UCP1 protein levels in SGBS adipocytes peak at Day 14 and are comparable to levels detected in differentiated PAZ6 cells. Lysates of SGBS and PAZ6 cells were prepared at D0, D14 and D28 (SGBS only) and immunoblotted with antibodies against UCP1. Equal protein loading was confirmed with antibodies against β-actin. CSC-C4-2 and CSC-LNCaP lysates are shown as positive control. CSC = cancer stem cells.

To determine protein levels of UCP1 in SGBS adipocytes we directly compared D14 and D28 samples with differentiated PAZ6 cells. Interestingly, UCP1 protein content in SGBS cells peaked at D14 and was comparable or slightly above levels detected in mature PAZ6 adipocytes (Figure 6). The abundance of UCP1 protein declined in SGBS adipocytes up to D28 as seen by immunofluorescence imaging (Figure 4) and measured on the mRNA level by quantitative RT-PCR (Figure 5). Two cancer stem cells samples, namely C4-2 and LNCaP, are shown as positive control of UCP1 expression as Valle et al. recently reported its abundance in several types of cancer [22].

#### Next-generation deep sequencing of three human adipocytes reveals pathways involved in the molecular and phenotype-switch observed in SGBS cells

**Figure 7:**
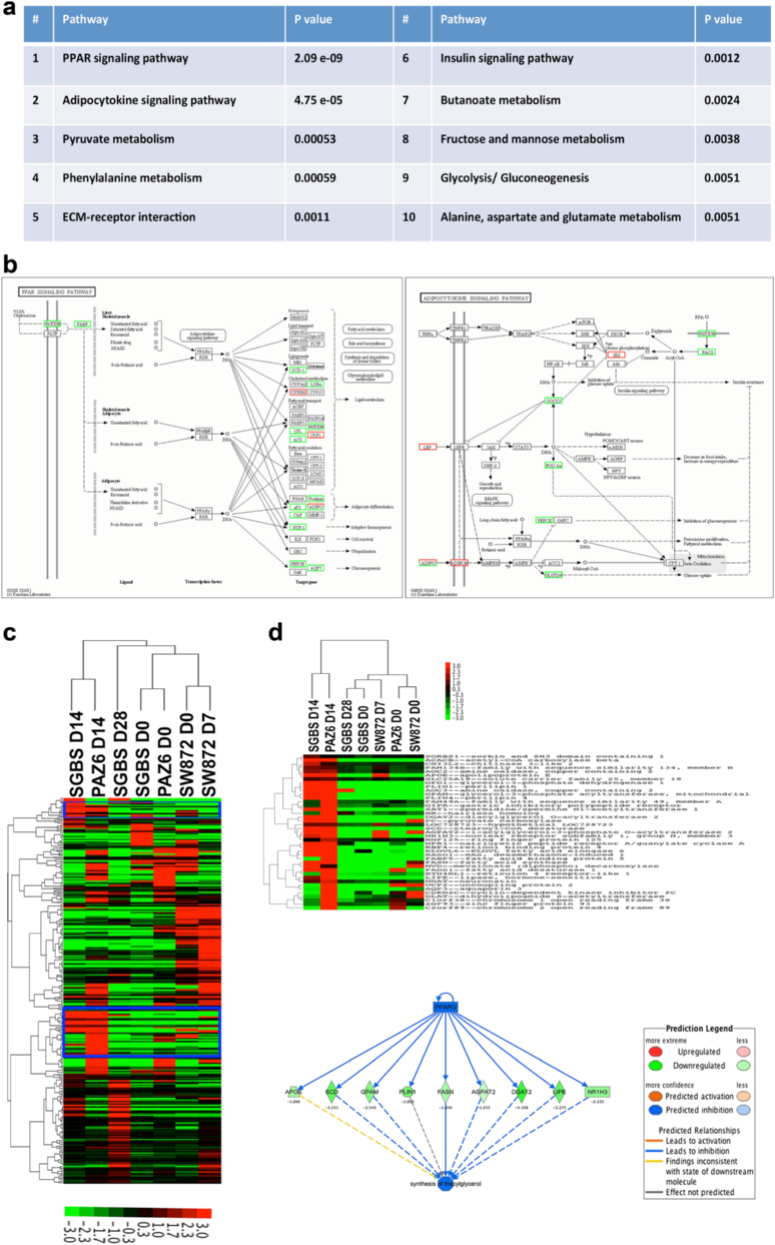
Next-generation whole genome RNA sequencing elucidates the activation of key pathways in three human adipocytes upon differentiation. RNA was isolated as described above and subjected to next generation sequencing analysis. Comparison of SGBS cells at D14 and D28 revealed 485 DEGs with fold change >2 and FDR <0.001. Genes were queried against know annotation databases and gene ontology analysis was performed. **(a)** 10 top-involved pathways are shown; among those PPARγ and adipocytokine signaling are the two most significantly involved pathways **(b). (c)** Expression levels of 284 DEGs with no absence calls between SGBS S14 and D28 samples were clustered for all three human adipocytes and visualized by Treeview software. Two clusters highlighted in blue are characteristic for SGBS D14, representative of the brownish stage, and differentiated, brown PAZ6 cells on D14. **(d)** 41 DEGs are displayed separately using Treeview software (upper panel). PPARγ gene interaction networks were generated with Ingenuity Pathway analysis software (lower panel). Log2 ratios are reported based on the comparison between SGBS D14 vs. SBGS D28.

To further elucidate the involvement of molecular pathways and molecules involved in the observed conversion of SGBS adipocyte from a brownish stage at D14 to a more WAT-like phenotype on D28 we performed next-generation RNAsequencing. SW872 cells were assessed at D0 and D7, PAZ6 adipocytes at D0 and D14 and lastly, SGBS cells were sequenced at D0, D14 and D28. Comprehensive bioinformatics analysis was performed which revealed 485 DEGs between the SGBS D14 and D28 sample with fold change >2 and FDR <0.001. Out of those transcripts 272 were found to be up-regulated and 213 were down-regulated at D28. We queried differentially expressed genes against known pathways by gene ontology analysis and the top 10 involved pathways are shown in Figure 7a. Interestingly, the pathway mostly affected by the phenotypic changes seen in differentiated SGBS cell is the PPARγ pathway followed by the adipocytokine signaling pathway. As shown in Figure 7b, many transcripts including UCP1 are down-regulated in SGBS D28 when compared to D14 suggesting derogated PPARγ signaling. In addition, the second most affected pathway is adipocytokine signaling (Figure 7a and b). Interestingly, though this pathway is involved in all types of adipocyte signaling and not specific for brown adipocytes, we observed a particular down-regulation of PGC1 α whilst leptin remained up-regulated. Taken together, these findings illustrate that key markers for BAT such as UCP1 and PGC1α are down-regulated in SGBS cells at D28 while no general affect on lipid metabolism was observed as seen by up-regulated leptin levels.

We then plotted the expression values for DEGs identified by comparing SGBS D14 with D28 along with the undifferentiated and differentiated samples of PAZ6 and SW872, respectively. Clustering analysis based on centered correlation was performed for the remaining 284 transcripts and data were displayed with Treeview software (Figure 7c). Interestingly, SGBS D14 and PAZ6 D14 samples clustered together suggesting common gene expression patterns. SGBS and PAZ6 pre-adipocytes formed another cluster as well as differentiated SW872, which clustered along with their undifferentiated counterpart. Further analysis of a small subset of genes, which is exclusively shared between SGBS D14 and PAZ14 samples (blue boxes, Figure 7c) is depicted in Figure 7d, and shown as a headmap image (upper panel). We subjected the 41 genes to pathway analysis and found even in this small subset of genes PPARγ as the top upstream regulator, which is significantly inhibited in SGBS D28, compared to SGBS D14 cells (p value 1.33E-28) along with nine downstream targets (namely ApoE, SCD, GPAM, PLIN1, FASN, AGPAT2, DGAT2, LIPE and NR1H3) which are shown in Figure 7d, lower panel.

#### Quantification of mitochondrial respiration rates of the PAZ6, SGBS and SW872 cell lines at different stages of differentiation

We have used the Luminex technology to obtain the relative expression of the mitochondrial respiratory complexes I, II, III, IV and V proteins and the SeaHorse XF24 and XF96 Flux analyzers to assess oxygen consumption and glycolysis in the three cell lines.

**Figure 8:**
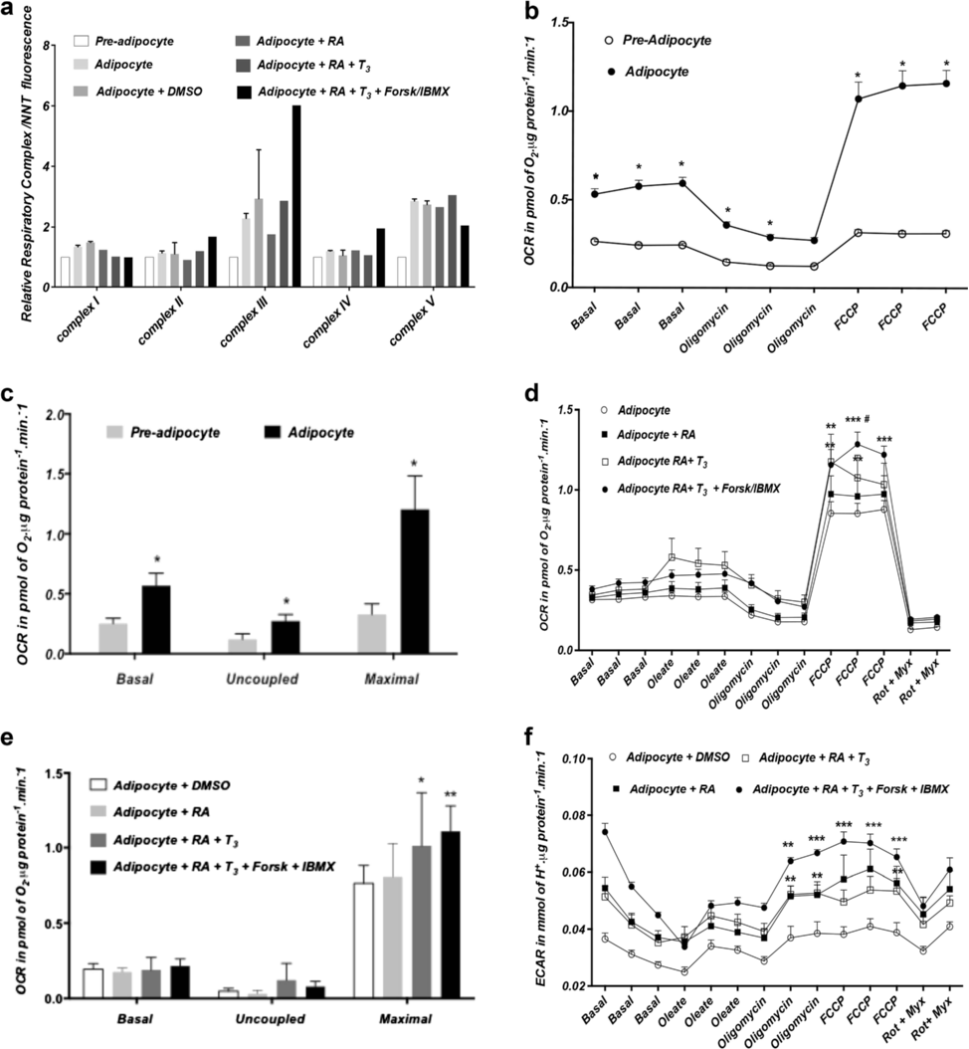
Metabolic characterization of undifferentiated and differentiated PAZ6 cells. **(a)** Expression of mitochondrial respiratory complexes I, II, III, IV and V in PAZ6 pre-adipocytes and adipocytes. Differentiation is associated with an overall increase in the expression of mitochondrial electron transfer chain complexes. Treatment of PAZ6 adipocytes with retinoic acid (1 μM), T_3_ (2nM) and Forskolin (2 μM)/IBMX (125 μM) further increases the expression of complexes II, III and IV. Values are presented as the fluorescent units ratio between each respiratory complex protein and the nuclear encoded NNT protein ± SEM. **(b)** OCR of PAZ6 pre-adipocytes and adipocytes in unbuffered DMEM containing 5 mM pyruvate and 2.5 mM glucose. **(c)** Basal, uncoupled and maximal respiratory capacities are robustly increased in the adipocyte relative to the pre-adipocyte state (* p < 0.0001). **(d)** OCR in PAZ6 adipocytes under various treatments (24 hrs).. FCCP-dependent (State3u) respiration is mildly increased in adipocytes treated with retinoic-acid (RA) plus T_3_ (**p < 0.05 vs. adipocyte). Forskolin/IBMX had a synergistic effect upon State3u respiration when given in combination with RA (***p < 0.0001 vs. adipocyte; # p < 0.05 vs. adipocyte + RA). **(e)** Basal, uncoupled and maximal respiration were calculated. Maximal respiratory capacity is increased in adipocytes treated with RA plus T_3_ (*p < 0.05 vs. adipocyte) and RA plus T_3_ plus forskolin/IBMX (**p < 0.05 vs. adipocyte). **(f)** Glycolysis, shown as the rate of extracellular acidification (ECAR) in PAZ6 pre-adipocytes and adipocytes under several conditions. Rates are significantly increased in adipocytes treated with RA, RA plus T_3_ (**p < 0.01 vs. adipocyte) and RA plus T_3_ plus forskolin/IBMX (***p < 0.001 vs. adipocyte). Results expressed as mean ± SEM.

**Figure 9:**
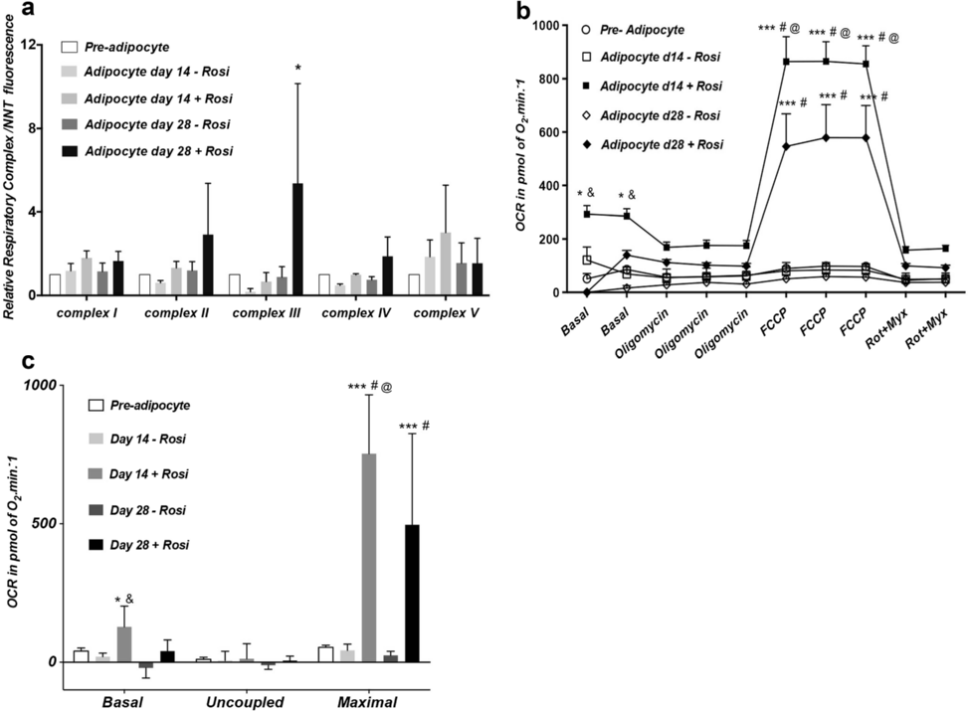
Metabolic characterization of undifferentiated and differentiated SGBS cells. Where noted rosiglitazone (rosi) was added during the first initial days of differentiation at a final concentration of 2 μM. **(a)** SGBS respiratory complexes expression. An overall increase in expression results from differentiation in the presence of rosiglitazone (*p < 0.02). Values are presented as the fluorescent units ratio between each respiratory complex protein and the nuclear encoded NNT protein ± SEM. **(b)** Oxygen consumption rate (OCR) by SGBS pre-adipocytes and adipocytes in unbuffered DMEM containing 5 mM pyruvate and 2.5 mM glucose. Basal and FCCP-dependent State3u respiration peaked at D14 in a rosi-dependent manner. **(c)** Basal, uncoupled and maximal respiration are shown. Basal respiration is increased in adipocytes at D14 in the presence of rosi (*p < 0.05 vs. adipocyte D14 – rosi; & p < 0.01 vs. pre-adipocyte). Maximal respiratory capacity is increased in the adipocytes differentiated in the presence of rosi at D14 and D28 (*** p < 0.0001 vs. pre-adipocyte, # p <0.0001 vs. adipocyte D14 or D28– rosi; @ p < 0.0001 vs adipocyte D28 + rosi). Results are expressed as mean ± SEM.

**Figure 10:**
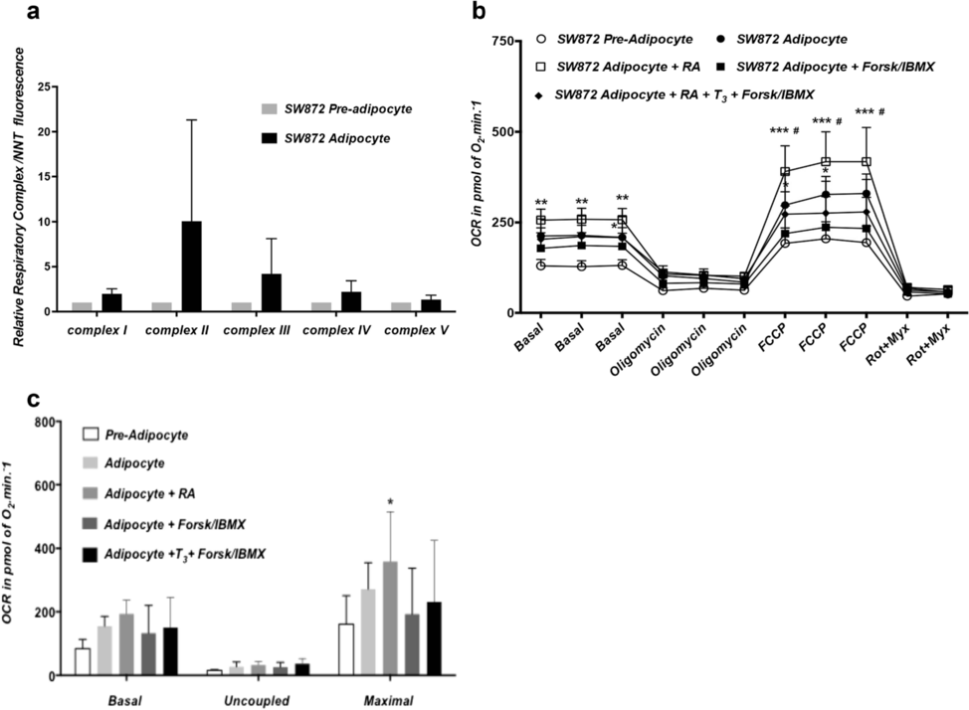
Metabolic characterization of undifferentiated and differentiated SW872 cells. **(a)** SW872 respiratory complexes expression. Differentiation in oleate increases the expression of the mitochondrial respiratory complexes. Values are presented as the fluorescent units ratio between each respiratory complex protein and the nuclear encoded NNT protein ± SEM. **(b)** OCR by SW872 pre-adipocytes and adipocytes in unbuffered DMEM containing 5 mM pyruvate and 2.5 mM glucose. Basal and FCCP-dependent State3u respiration are increased in the RA-treated adipocyte relative to the pre-adipocyte state (** p < 0.001; ***p <0.0001 versus pre-adipocyte; # p <0.05 vs. adipocyte + RA + T_3_ + forskolin/IBMX)). **(c)** Basal, uncoupled and maximal respiration are calculated. RA increased the maximal respiratory capacity of the adipocytes relative to the pre-adipocytes (*p < 0.05). Results expressed as mean ± SEM.

Differentiation of PAZ6, SGBS and SW872 promoted an overall induction of respiratory complex proteins relative to the nuclear-encoded protein NNT (Figures 8a, 9a and 10a). In line with this increase in respiratory complex proteins expression, the oxidative metabolic capacity was increased in the adipocyte relative to the pre-adipocyte state in all three lines. A robust raise in State3u, FCCP-dependent respiration was observed as a result of PAZ6 differentiation (Figure 8b). Basal, uncoupled and maximal respiratory capacity, obtained after rotenone/myxothiazol-dependent non-mitochondrial respiration were higher in the differentiated PAZ6 cells compared to their undifferentiated counterparts (Figure 8c), Twenty-four hours treatment with a retinoic acid (RA), T_3_ or RA, T_3_ and forskolin/IBMX (FI) cocktail further induced complexes II, III and IV (Figure 8d) and increased the maximal respiratory capacity of PAZ6 adipocytes (Figure 8e). Notably, this combined treatment also significantly promoted a raise in the rate of extracellular acidification of PAZ6 adipocytes, indicating an increase in glycolytic capacity (Figure 8f). The induction of glycolysis by RA, T_3_ and FI combined was higher than the induction observed upon RA treatment alone or in combination with T_3_ only (Figure 8f).

In SGBS cells, the induction of mitochondrial respiratory proteins and oxidative capacity in adipocytes required rosiglitazone and was dependent on differentiation length (Figure 9a). Whereas complex I had comparable levels of expression at D14 and D28 in the presence of rosiglitazone, complex III was higher at D28 while complex V was higher at D14 (Figure 9a). State 3u, FCCP-dependent respiration peaked at D14 although it still remained elevated at D28 relative to the undifferentiated cells (Figure 9b). Calculated basal and maximal respiration were the highest at D14 (Figure 9c).

In SW872 cells, the increase in respiratory complexes was observed for all five complexes in the differentiated adipocytes (Figure 10a). This increase was accompanied by a matching increase in State 3u, FCCP-dependent maximal oxidative capacity in the differentiated compared to the undifferentiated state. RA alone promoted an additional increased in maximal respiratory capacity (Figure 10b) and forskolin/IBMX did not synergize with RA in improving respiration but instead trended towards suppressing it (Figure 10c).

#### Cold exposure of SGBS adipocytes activates BAT markers and leads to an enhanced brown adipocyte phenotype

**Figure 11:**
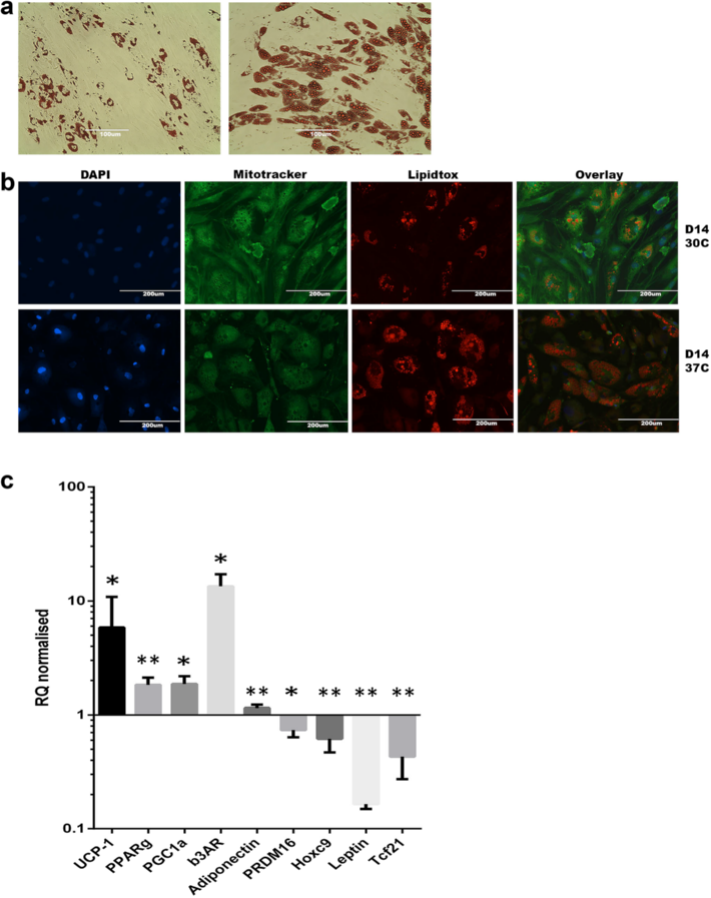
Cold exposure of SGBS adipocytes activates BAT markers and leads to an enhanced brown adipocyte phenotype. SGBS cells were cultured at 30°C for either 10 days or 4-6 h and objected to phenotypic or molecular assessment of BAT and WAT markers. **(a)** SGBS cells were transferred to cold environment after the first 4 days of initial differentiation and compared to control cells which were kept at 37°C for the duration of the two week differentiation process. Oil Red staining was carried out as described above and the presence of stained lipid droplets at D14 was assessed. **(b)** 10 day cold-exposed and control SGBS cells were co-stained with mitotracker (green) to test the abundance of mitochondria, lipidtox (red) for neutral lipid droplets and DAPI (blue) to visualize nuclei. Single-channel images were overlayed and processed by Photoshop software. All scale bars are reported. **(c)** RNA from SGBS cells, which were subjected to cold-environment for 4-6 h, was isolated using the Qiagen lipid tissue mini kit and cDNA synthesis was carried out. Quantitative real-time PCR was performed by SYBR green detection method and expression levels are normalized for D0 baseline expression levels. All experiments were done in triplicates and data are derived from at least three independent experiments. (* p <0.01, ** p < 0.001).

Lastly, we explored the capacity of SGBS cells to respond to cold cell-autonomously (in a β-adrenergic-independent manner) to see whether the brownish phenotype seen at D14 can be further enhanced. Indeed, we noticed an increase in lipid droplet number and decrease in droplet size when SGBS cells were cultured at 30°C for 10 days following initial differentiation at 37°C for four days and compared to the control cells which were kept entirely at 37°C (Figure 11a and b). This finding was observed through Oil Red staining (Figure 11a), and by fluorescence staining of lipid droplets (Figure 11b) and UCP1 expression (Figure 11c). Of note, UCP1 expression and mitotracker co-staining were elevated in the cold-exposed SGBS cells and underlined the inducible brown phenotype. We further assessed the molecular expression level of known adipocyte markers of SGBS cells that were transferred to 30°C for 6 h compared to control cells at D14. Notably, expression levels of UCP1, PPARγ, PRDM16 were significantly increased in cells exposed to a cold environment wheras no further increase in the expression of WAT markes such as Tcf21 and Hoxc9 were found (Figure 11c).

## Discussion

It is common wisdom that obesity and its associated metabolic and inflammatory complications have become one of the top risk factors for mortality, long term disability and financial burden to modern societies worldwide [23,24].

The lack of efficient treatment options available to the medical community urgently calls for scientists to bring meaningful and innovative ways to combat this pandemic. The in-depth understanding of the biology of adipose tissues is a critical piece to solving this obesity puzzle. Beyond its originally defined fat storage function, adipose tissue exhibits endocrine gland-like functions, secreting mediators that exert behavioral, metabolic and inflammatory actions at distant organs. Its non-parenchymal cells contribute to the synthesis of immune mediators with pro-inflammatory properties [25]. Furthermore, the highly specialized brown adipose sub-type displays the intrinsic ability to generate heat through a process called non-shivering thermogenesis (NST), which confers mammals the ability to maintain body temperature constant despite that of the environment [26]. Due to its substantial caloric cost, NST has begun to be explored therapeutically in the past seven years [26]. It is believed that NST can be beneficial in the overweight or obese scenarios, by counteracting the excessive energy and fat deposition which constitute the main risk factors for cardiovascular disease, T2D, chronic inflammation and cancers.

Though adipose tissue can be studied *in vivo*, the integrative nature of metabolism complicates the understanding of the specific role it plays in the whole animal. Each time a new genotype or compound effects is tested, most metabolic parameters are gathered at defined endpoints so the phenotypes or drug-effects observed reflect the sum of the role played by all tissues combined. This is an extremely valuable approach and a key step in the drug development pipeline. Nevertheless, complementing such *in vivo* findings with studies in cell models allows for the delineation of the cellular mechanisms behind the effects of the genetic and chemical interventions in question upon the whole animal biology. *In vitro* studies obviously also allow for much quicker and less costly preliminary testing that can unravel drug and protein targets to be pursued.

With this in mind, we were prompted to extend the characterization of the unique immortalized human pre-brown adipocyte cell line PAZ6 [27], aiming that its full potential can be utilized in metabolic research. Furthermore, we have paralleled our study of PAZ6 cells with that of two other human pre-adipocytes cells lines, the SGBS and the SW872 cells, in order to cover the broad spectrum of adipocyte phenotypes, which ultimately impact metabolic outcomes.

A few other strains of human white adipocytes, whose characterization is beyond the scope of this study, are available. The LiSa-2 [28], LS-14 [29] and AML-1 [30] cell lines have originated from spontaneous tumors and the Chub-S7 line was immortalized by means of telomerase reverse transcriptase and papillomavirus E7 oncoprotein transformation [31]. With regards to brown pre- or adipocyte cell lines, there are no other immortalized human cell lines to our knowledge. However, human multipotent adipose-derived stem cells (MADS) that have the potential to differentiate in either white or brown fat adipocytes have been established in culture [32]. Additionally, SVF from supra-clavicular fat [33] and primary stem cells [34] have also been utilized in studies of human brown adipose in vitro. A comparison between the PAZ6 features and those of MADS should be informative in the future.

By extensively comparing and characterizing three human adipocyte cell lines we noticed that the SGBS cell line harbors a versatile phenotype, which changes throughout its mature stage and questions the characterization of SGBS as a true white adipocyte cell line model. D14 displays multiple characteristics of brown fat cells such as UCP1 and PPARγ overexpression whereas D28 represents a rather white phenotype.

Jo et al. previously reported the existence of a temporary increase of PPARγ and transient expression of UCP1 in the stromal-vascular fraction of adipose-derived stem cells (ADSCs), which were induced to differentiate [35]. We also considered the possibility that the induction of brown-adipocyte-like features upon differentiation in cell line settings was induced artificially since some studies reported that chronic treatment of white adipocytes with the peroxisome proliferator-activated receptor γ agonist rosiglitazone promotes PGC1α and UCP1 expression along with an increase in mitochondriogenesis [36]. In our study, PAZ6 and SGBS differentiation media were also enriched with rosiglitazone, as this is a standard formulation and widely used. We therefore depleted rosiglitazone from the SGBS medium to investigate whether our observation was mainly attributed to its presence. Nevertheless, even in absence of rosiglitazone we noticed up-regulation of BAT markers such as UCP1 and PPARγ in SGBS cells at D14, which then declined up to D28 (data not shown). We therefore conclude that we are not promoting the phenotype observed in maturating SGBS cells by drug treatment, but rather are seeing an intrinsic property of the SGBS adipocyte cell line itself.

The third cell line, which we characterized, was SW872. Even though SW872 cells are widely used as a human pre-adipocyte model [37] some features are not fully characteristic for WAT. We noticed that the differentiation of SW872 cells could be initiated at confluency levels less than 100% which was indeed required for PAZ6 and SGBS cells. Similarly, we observed that 100% of SW872 cells were fully differentiated at D7 by applying the above described culture conditions. Additionally, SW872 cells continued to proliferate even during the differentiation process and did not undergo contact inhibition and growth arrest (data not shown). Of special note, we observed that differentiated SW872 adipocytes displayed many characteristics of WAT, however especially lipid droplet size and numbers were not characteristic for the white adipocyte phenotype.

In addition to the up-regulation of characteristic BAT markers in SGBS and PAZ6 cells, we noticed high levels of adiponectin in mature PAZ6 and SGBS cells at D14. Notably in line with the transient phenotype, adiponectin levels in SGBS cells decrease up to D28 by 50% as opposed to leptin levels. Interestingly, adiponectin is known for its anti-inflammatory properties and inverse correlation to insulin resistance and its levels in humans rise in response to weight loss and exercise [38-40]. Next generation sequencing revealed that 5 out of the top 10 pathways, which are differentially expressed between SGBS cells at D14 and D28, are involved in adipogenesis and/or metabolism. Among them the PPARγ pathway followed by the adipocytokine signaling pathway are most significantly affected. PPARγ is a member of the nuclear receptor superfamily of ligand-inducible transcription factors which control the expression of genes mainly involved in adipogenesis, lipid metabolism, inflammation and maintenance of metabolic homeostasis. Interestingly, PPARγ signaling was significantly and exclusively up-regulated in PAZ6 and SGBS cells at Day 14, but underwent inhibition in SGBS cells cultured to D28. Furthermore, many downstream molecules such as UCP1 were down-regulated in SGBS cells at D28, which indicates the shift from the brown to the white phenotype. Interestingly, Perilipin 1 (PLIN1) was also among the genes down-regulated in SGBS cells at D28. A recent study from Sawada et al. revealed that overexpression on PLIN1 lead to an induction of a brown-like or beige phenotype in the WAT of transgenic mice [41], which we noticed in our study in SGBS at D14 when PLIN1 expression peaked.

Furthermore, our delineation of the expression of mitochondrial complexes and metabolism in these three adipocyte cell lines corroborates our genomics and proteomics results and fulfills the demand for functional assessments in metabolic cell models. The generally increased levels of respiratory complexes were expectedly matched by improved respiratory capacity in adipocytes relative to their undifferentiated counterparts. We believe this is an interesting finding and the reason is two-fold: firstly, the differentiation process of all three cell lines was effective in launching their oxidative capacity. This is significant in the *in vitro* context because cells in culture normally shift their metabolism towards anaerobic glycolysis, which was indeed predominant in the pre-adipocyte but not in the differentiated adipocyte state. Secondly, adipose tissue is generally regarded as metabolically slow. However, both differentiated PAZ6 and D14 SGBS cells are shown to dispose of spare respiratory capacities several fold greater than their undifferentiated counterparts. Whether this is an intrinsic property of the differentiated cells, an effect exclusively dependent on the differentiation cocktail or both, remains to be defined. It would be extremely informative to address how the metabolic capacity of PAZ6 and SGBS compares to that of other human tissues on a per mg of protein basis.

As for drug effects, previously reported effects of RA, T3 and forskolin/IBMX in promoting a brown adipose-like phenotype [42] were observed in PAZ6 and SW872 adipocytes in this study by means of induction of respiratory complexes and oxidative capacity. The effect of the RA, T3 and adrenergic signaling mimetics (IBMX/forskolin) upon glycolysis is an interesting finding. At this point we cannot dissociate the individual effects of each compound or their interactions with the differentiation cocktail. However, it seems plausible that in addition to boosting lipid oxidation, brown adipose tissue may also utilize glycolysis to support its metabolic demands. Recently, Hao et al. described the global expression changes is interscapular BAT (iBAT) of cold exposed C57 mice [43]. This *in-vivo* experiment was complemented with the *in-vitro* treatment of primary brown adipocytes with isoproterenol for 6 hours. In both cases, an increase in several glycolytic enzymes was detected, supporting the notion that glycolysis may be a relevant component in BAT cold adaptation. In line with this, human imaging studies employing F-DG as a BAT activity marker consistently describe increased F-DG uptake in cold-exposed individuals. Whether the incoming glucose is utilized anaerobically or shunted to supply uncoupled respiration needs to be determined. Whatever the case, this improved capacity for glucose utilization observed in treated PAZ6 adipocytes, provides a means whereby brown adipocytes could scavenge excess circulating glucose, which could be explored to improve glucose homeostasis in diabetic states.

## Conclusions

Overall, our study investigates intrinsic properties of the unique human brown adipose cell line PAZ6, human white SW872 adipocytes and human SGBS cells that display a transient brown phenotype which can be further induced by β-adrenergic stimulation through cold exposure. Even though this behavior was shown in only one cell line and cannot be generalized at this point, our unique study contributes to the discovery of molecular gene expression patterns and pathways, which are involved in the conversion from white and brown adipocytes. This knowledge will be of importance for translational studies aimed at increasing brown fat depots in order to increase energy expenditure in obese subjects with the ultimate goal of weight reduction.

## Additional file

Additional file 1: Table S1. List of primers and their sequences used in quantitative RT-PCR.

## Abbreviations

BAT: Brown adipose tissue
IBMX: 3-isobutyl-1-methylxanthine
UCP1: Uncoupling protein 1
WAT: White adipose tissue
SGBS: Simpson-Golabi-Behmel gigantism
PPARγ: Peroxisome Proliferator Activated Receptor
BMI: Body mass index
T2D: Type 2 diabetes
SirT1: Sirtuin 1
PGC1α: Peroxisome proliferator activated receptor gamma coactivator
PRDM16: PR domain zinc finger protein 16
b3AR: Beta3-adrenergic receptor
NNT: Nicotinamide nucleotide transhydrogenase
ATCC: American Type Culture Collection
OCR: Oxygen consumption rate
ECAR: Extracellular acidification rate
NST: Non-shivering thermogenesis
MADS: Multipotent adipose-derived stem cells
ADSC: Adipose-derived stem cells
PLIN1: Perilipin 1
Tcf21: Human Transcription factor 21
RT: room temperature
GO: Gene ontology
DEGs: Differentially expressed genes
GEO: Gene Expression Omnibus
NCBI: National Center for Biotechnical Information
FU: Fluorescent units
