# Supplementary material for: Comprehensive molecular characterization of human adipocytes reveals a transient brown phenotype
Source: J Transl Med. 2015 Apr 30;13:135. doi: 10.1186/s12967-015-0480-6 (PMC4438513; doi:10.1186/s12967-015-0480-6)
Supplement: Additional file 1: Table S1. — List of primers and their sequences used in quantitative RT-PCR. [file 12967_2015_480_MOESM1_ESM.pdf]

Comprehensive molecular characterization of human adipocytes reveals a transient brown phenotype

Andrea Guennoun[1], Melissa Kazantzis[2], Remy Thomas[1], Martin Wabitsch[3], Daniel Tews[3], Konduru S. Sastry[1], Mouaadh Abdelkarim[4], Vladimir Zilberfarb[5]†, A. Donny Strosberg[6]† and Lotfi Chouchane[1*]

[1] Laboratory of Genetic Medicine & Immunology, Weill Cornell Medical College in Qatar, Doha, Qatar

[2] Center for Diabetes and Metabolic Diseases, The Scripps Research Institute, Florida, USA

[3] Department of Paediatrics and Adolescent Medicine, Division of Pediatric Endocrinology and Diabetology, Ulm, Germany

[4] Department of Physiology, King Saud University, Riyadh, Saudi Arabia

[5] Institut Cochin INSERM U1016, Université Paris 7-Denis-Diderot, Paris, France

[6] Department of Infectology, The Scripps Research Institute-Florida, Jupiter, FL, USA

† Deceased

* Corresponding author:

Prof. Lotfi Chouchane, Weill Cornell Medical College in Qatar, P.O. Box 24144, Doha, Qatar. Email: loc2008@qatar-med.cornell.edu

Table S1. *Primer sequences for quantitative RT-PCR analysis*

**Gene Name**	**Forward**	**Reverse**
HPRT	tgacactggcaaaacaatgca	ggtccttttcaccagcaagct
UCP1	tctctcaggatcggcctcta	ccgtgtagcgaggtttgatt
PRDM16	tcctgaagacattccgatcc	ccgaagtctgtctcctttgc
b3AR	cagtggtgccttacatggtg	tgggaaggtagaggttgtgg
PPARγ	tccatgctgttatgggtgaa	tcaaaggagtgggagtggtc
PGC1α	cctgcatgagtgtgtgctct	gcaaagaggctggtcttcac
Perilipin	ctctcgatacaccgtgcaga	tggtcctcatgatcctcctc
Leptin	ggctttggccctatcttttc	ccaaaccggtgactttctgt
Adiponectin	cctaagggagacatcggtga	gtaaagcgaatgggcatgtt
Tcf21	gaaacccgagagtgacctga	tggtttggaaacgaatctcc
Hoxc9	acgtggactcgctcatctct	cacgacgtgctgaacactg
